# Identification of Putative Candidate Genes for Water Stress Tolerance in Canola (*Brassica napus*)

**DOI:** 10.3389/fpls.2015.01058

**Published:** 2015-11-27

**Authors:** Jing Zhang, Annaliese S. Mason, Jian Wu, Sheng Liu, Xuechen Zhang, Tao Luo, Robert Redden, Jacqueline Batley, Liyong Hu, Guijun Yan

**Affiliations:** ^1^Ministry of Agriculture (MOA) Key Laboratory of Crop Ecophysiology and Farming System in the Middle Reaches of the Yangtze River, College of Plant Science and Technology, Huazhong Agricultural UniversityWuhan, China; ^2^Centre for Plant Genetics and Breeding, School of Plant Biology, Faculty of Science and The UWA Institute of Agriculture, The University of Western AustraliaPerth, WA, Australia; ^3^Plant Breeding Department, IFZ Research Centre for Biosystems, Land Use and Nutrition, Justus Liebig UniversityGiessen, Germany; ^4^School of Agriculture and Food Sciences and Centre for Integrative Legume Research, The University of QueenslandBrisbane, QLD, Australia; ^5^National Key Laboratory of Crop Genetic Improvement, College of Plant Science and Technology, Huazhong Agricultural UniversityWuhan, China; ^6^Australian Grains Genebank, Department of Economic Development Jobs Transport and ResourcesHorsham, VIC, Australia

**Keywords:** canola, water stress tolerance, genome-wide association study, RNA sequencing, drought-related genes

## Abstract

Drought stress can directly inhibit seedling establishment in canola (*Brassica napus*), resulting in lower plant densities and reduced yields. To dissect this complex trait, 140 *B. napus* accessions were phenotyped under normal (0.0 MPa, S0) and water-stressed conditions simulated by polyethylene glycol (PEG) 6000 (−0.5 MPa, S5) in a hydroponic system. Phenotypic variation and heritability indicated that the root to shoot length ratio was a reliable indicator for water stress tolerance. Thereafter, 66 accessions (16 water stress tolerant, 34 moderate and 16 sensitive lines) were genotyped using 25,495 *Brassica* single nucleotide polymorphisms (SNPs). Genome-wide association studies (GWAS) identified 16 loci significantly associated with water stress response. Two *B. napus* accessions were used for RNA sequencing, with differentially-expressed genes under normal and water-stressed conditions examined. By combining differentially-expressed genes detected by RNA sequencing with significantly associated loci from GWAS, 79 candidate genes were identified, of which eight were putatively associated with drought tolerance based on gene ontology of *Arabidopsis*. Functional validation of these genes may confirm key drought-related genes for selection and breeding in *B. napus*. Our results provide insight into the genetic basis of water stress tolerance in canola.

## Introduction

Canola (*Brassica napus*) is one of the largest oil crops in the world together with soybean and oil palm. However, it is very sensitive to drought stress, a major yield-limiting factor in this crop (Wan et al., 2009). Drought stress can directly inhibit seedling establishment, resulting in lower plant densities and reduced yields. Increased drought conditions are predicted worldwide due to the predicted long-term effects of global climate change (Cook et al., 2007). An important solution to this challenge is the development of canola cultivars which can tolerate water stress in order to maintain oil production. A key step toward genomics-assisted breeding for water stress tolerance in canola involves characterization of functional water-stress-tolerant genes or markers closely linked to these genes (Xue et al., 2013).

Genetic improvement of crops for drought tolerance requires investigation of possible mechanisms including water stress tolerance at the seedling stage and exploration of genetic variation for drought tolerance within a species (Dhanda et al., 2004; Lu et al., 2011). Previous studies have revealed considerable variation in water stress tolerance in wheat (Dhanda et al., 2004), sorghum (Bibi et al., 2012) and soybean (Bouslama and Schapaugh, 1984; Kpoghomou et al., 1990). In wheat seedlings, Dhanda et al. (2004) indicated benefits from selection for osmotic membrane stability of leaf segments and root to shoot length ratio. In sorghum seedlings, root length had the highest proportional contribution to drought tolerance (Bibi et al., 2012). In soybean, plant height stress index was a reliable parameter for predicting cultivar growth performance in early stages of development (Kpoghomou et al., 1990). Thus, seedling characteristics can often be used to study the effects of water stress on plants and to identify underlying functional genes.

Genome-wide association studies (GWAS) have proven powerful in revealing the complex genetic basis of important traits in sequenced genomes of *Arabidopsis thaliana*, rice and maize (Atwell et al., 2010; Huang et al., 2010, 2012; Zhao et al., 2011; Wang et al., 2012; Li et al., 2013; Chen et al., 2014; Wen et al., 2014; Matsuda et al., 2015; Ogura and Busch, 2015). Single nucleotide polymorphisms (SNPs) are currently recognized as the marker of choice in most species for GWAS (Raman et al., 2014). Few GWAS-based studies had been reported in canola until recently, due to limited marker numbers, low-throughput genotyping technologies and few available lines (Cai et al., 2014; Li et al., 2014a). However, the recent release of the Illumina Infinium Brassica 60K SNP array produced by the International Brassica SNP Consortium, and the sequenced and annotated genome of *B. napus* (http://www.genoscope.cns.fr/brassicanapus) (Chalhoub et al., 2014), now provide a low-cost and efficient method for high-density, sequence-based, genome-wide polymorphism screening in *B. napus* populations (Liu et al., 2013; Raman et al., 2014). Since these advances, GWAS has been used to dissect the genetic control of seed weight and quality, seed germination and seedling vigor in *B. napus* (Li et al., 2014a; Hatzig et al., 2015). Although the genetic mechanisms of drought tolerance are complex, GWAS has been successfully used to identify functional variation in both known and unknown genes associated with drought tolerance in maize (Hao et al., 2011; Xue et al., 2013). Previous drought studies in canola only used low marker density QTL mapping, which may be unable to capture global genetic diversity for drought tolerance (Li et al., 2014b; Fletcher et al., 2015).

In this study, 140 canola accessions were phenotyped under normal and water stress (simulated by PEG 6000) conditions; of these, 66 accessions ranging from water stress tolerant through to sensitive were selected to perform GWAS using the Illumina Infinium Brassica 60 K SNP array (http://illumina.com; 52157 SNPs). Two selected accessions were used to conduct RNA sequencing (RNA-seq). The objective of this study was to identify SNP markers and candidate genes significantly associated with water stress tolerance during early seedling growth in canola.

## Materials and methods

### Plant materials

In this study, 140 accessions originating from 17 countries were screened. Most of the lines came from the Australia Temperate Field Crops Collection (ATFCC), while three came from Huazhong Agricultural University (HZAU) and six came from the Oil Crops Research Institute, Chinese Academy of Agriculture Science (OCRI-CAAS) (Supplementary Table 1).

### Experimental design and phenotyping

Seeds were germinated in petri dishes on filter paper (Whatman™, Malaga, WA, Australia) soaked with 5 ml deionized water and maintained in a dark incubator at 25°C. When the radicles were 3–5 mm long, seeds from each accession were transferred to two hydroponic boxes with deionized water. The seedlings were grown under controlled conditions in a constant temperature room (25°C) with 12 h light (93.7 μmol·m^−2^s^−1^) and 12 h dark at 50% relative humidity. After 5 days, seedlings were exposed to either 0.0 MPa (normal condition, S0) or −0.5 MPa (water stress, S5) for 7 days using ¼ strength Hoagland's solution or ¼ strength Hoagland's solution supplemented with polyethylene glycol (PEG) 6000 (Michel and Kaufmann, 1973; Michel, 1983), respectively. Deionized water was added to the solution to compensate for evaporation and the solution was stirred to ensure aeration. The experiment was arranged in a completely randomized design with five replications for each line in both treatments. Root length (RL), shoot length (SL), root to shoot length ratio (R/S) and fresh weight (FW) were recorded. Drought stress index (DSI) was calculated for each accession, $\text{DSI}=\frac{\text{FW under S}5}{\text{FW under S}0}\times 100\%$ (Kpoghomou et al., 1990).

### SNP genotyping and filtering

Based on the DSI, 66 inbred lines classified as water stress tolerant (>50%), moderate response (30–50%) or sensitive (< 30%) were selected and genotyped using the Illumina Infinium Brassica 60 K SNP array (Supplementary Table 2). DNA was extracted according to methodology detailed in Fulton et al. (1995). All DNA samples were hybridized to an Illumina Infinium Brassica 60 K SNP array released for the *B. napus* genome (http://illumina.com; 52157 SNPs) according to the manufacturer's instructions. SNP chips were scanned using an Illumina HiScanSQ and data visualized using Genome Studio V2011.1 (Illumina, Inc., San Diego, CA, USA) (Mason et al., 2015). The source sequences of the SNP array were used to perform a BLAST (Altschul et al., 1990) search against the public *B. napus* genome sequence (Chalhoub et al., 2014). Only the top blast hits were considered, with those matched to multiple loci not considered (Li et al., 2014a). SNPs with a call frequency < 0.8 or minor allele frequency (MAF) < 0.05 were excluded from further analysis. In addition, SNPs with >0.25 heterozygous calls were removed.

### Genome-wide association studies (GWAS)

The 66 genotyped lines were used for the GWAS association panel. The 4794 SNPs which were evenly distributed across the whole genome were selected to perform the relative kinship analysis using the software package SPAGeDi (Hardy and Vekemans, 2002). The relative kinship matrix (K) comparing all pairs of 66 lines was calculated. The software STRUCTURE was used to infer the population structure (Q) (Pritchard et al., 2000). Principal components analysis (PCA) was performed using GCTA tools (Yang et al., 2011). GWAS was performed using six models: naïve, Q, PCA, K, Q+K and PCA+ K in Tassel 4.0 (Bradbury et al., 2007; Li et al., 2014a). The model which fitted the data best was used to conduct GWAS with 25,495 SNPs (Supplementary Table 2) for data from each environment (S0 and S5). Significance was defined at a uniform threshold of *P* < 3.92 × 10^−5^ (*P* = 1/N, N = total markers used: Bonferroni-correction) (Wen et al., 2014).

### RNA extraction and sequencing

Whole seedlings of two drought sensitive inbred lines according to the drought stress index (Supplementary Table 1)—L72 (MUTU) and L106 (Alku)—were sampled after being subjected to S0 and −1.0 MPa (S10) for 6 h (methods as per Section Experimental Design and Phenotyping). Total RNA was extracted using a Bioline Isolation II RNA Plant Kit (Bioline, Sydney, NSW, Australia) and the genomic DNA contamination was removed by on-column digestion with DNase I which was included in the kit following the manufacturer's protocols. The yield was assessed using a NanoDrop™ ND-1000 spectrophotometer. The RNA quality was also assessed using agarose gel electrophoresis and Agilent 2100 to check the integrity and the residual DNA.

RNA from four samples was used to construct cDNA libraries separately with the Illumina® TruSeq™ RNA Sample Preparation Kit v2. The libraries were sequenced on an Illumina Hiseq™ 2000 platform following the manufacturer's protocols, which produced 50 bp paired-end reads. The raw reads were cleaned using the NGS QC toolkit to remove reads containing primer/adaptor sequences, low-quality reads (sequences whose number of bases with PHRED-like score (Q-score) less than 20 accounted for more than 30%) and reads less than 50 bp in length. Sequences with the first 10 base pairs of reads showing unstable base composition according to the percentages of four different nucleotides (A, T, C, and G) and low-quality reads (Q-score < 20) from 3′ end were also trimmed (Patel and Jain, 2012). All high-quality reads from each sample were separately mapped to the *B. napus* genome sequence (Chalhoub et al., 2014) by Tophat v2.0.11 using default parameters (Trapnell et al., 2009). Only uniquely-mapped reads were considered for gene expression analysis. The transcript abundance of each gene was estimated by FPKM (Fragments per Kilobase of transcript per Million fragments mapped) with a cutoff value of 1.

### Gene ontology and differential gene expression analysis

All *B. napus* genes (101,040) were searched against the National Center for Biotechnology Information (NCBI) non-redundant (Nr) protein database using BlastP with *E*-value ≤ 1E-05. Gene ontology (GO) terms associated with each BLAST hit were annotated using Blast2go (Conesa et al., 2005), and *B. napus* genes were searched against the InterPro database (http://www.ebi.ac.uk/interpro/) using InterProScan5 (Jones et al., 2014). The GO terms for the *B. napus* genes were annotated by merging Blast2go and InterPro annotation results. Furthermore, homologs of *Arabidopsis* genes in the *B. napus* genome were identified based on BlastP program with *E*-value ≤ 1E-05, identity ≥50% and coverage ≥50%. Genes that had a different transcript level between S0 and S10 samples were identified at | log_2_ (S10/S0)| ≥ 1 and false discovery rate (FDR) ≤ 0.01 across the two plant accessions assessed, and were subsequently regarded as differentially-expressed genes (DEGs).

### Real-time quantitaitve RT-PCR

The same RNA for RNA sequencing was used to obtain first-strand cDNA with the EasyScript First-strand cDNA Synthesis SuperMix kit (TransGen Biotech, Beijing, China) according to the manufacturer's instructions. Specific primers were designed for the target genes (Supplementary Table 5) using Primer 3 online software version 4.0.0 (Untergasser et al., 2012) and adjusted by oligo software version 7.56 (Rychlik, 2007). Real-time quantitative PCR (qRT-PCR) was performed in a 20 μL reaction vessel containing 10 μL *TransStart*® Tip Green qPCR Supermix (TransGen Biotech, Beijing, China), 10 pmoL of forward and reverse gene-specific primers, and 1 μL cDNA. PCR amplification was performed in an IQ5 machine (Bio-Rad, Hercules, CA, USA) according to the manufacturer's instructions with three independent technical replicates for each sample. Following a denaturation step at 95°C for 5 min, the amplification step comprised 40 cycles at 95°C for 15 s and 60°C for 30 s. A melting curve was constructed to determine the specificity of each PCR primer by maintaining the reaction at 95°C for 1 min, cooling the sample to 55°C for 1 min and finally heating to 95°C at a rate of 0.5°C per 6 s. The samples were initially normalized to a selected internal control gene (serine/threonine-protein phosphatase PP2A-1 catalytic subunit, *PP2A-1*), and the relative gene expression levels were determined using the 2^−ΔCt^ method (An et al., 2015).

### Data analysis

Analyses of variance and correlation coefficients were conducted using Statistix 8.1. Mean values of different characters were used to compute heritability (Burton and Devanez, 1953). QQ plots and Manhattan plots, Venn diagrams and heatmaps were drawn with gplots package, Vennerable and the pheatmap library, respectively, using R version 3.1.1 (http://www.r-project.org/) (R Foundation for Statistical Computing, 2012).

## Results

### Phenotypic variation and heritability of measured traits under normal (S0) and water stress (S5) conditions

**Figure 1:**
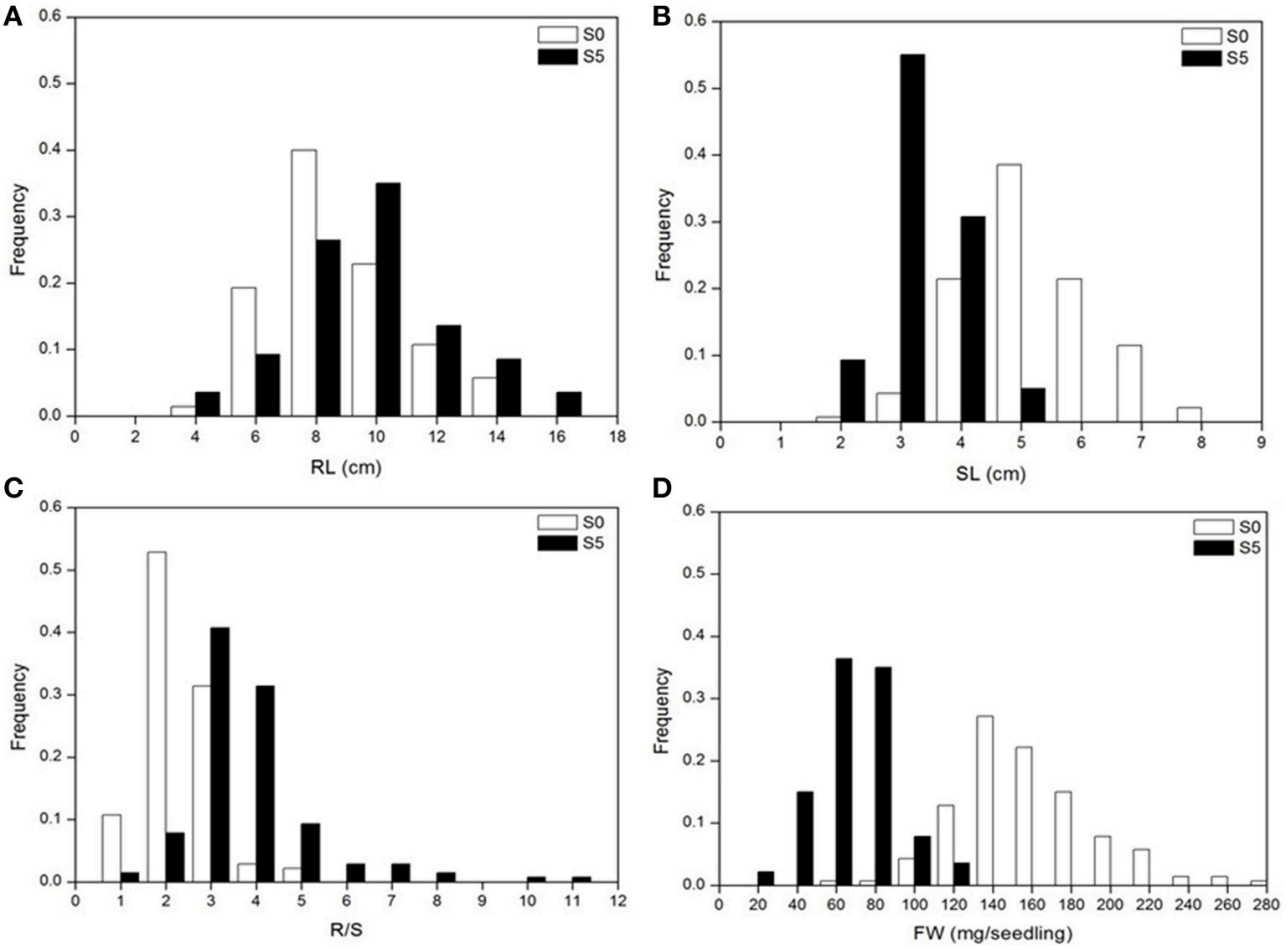
**Distribution of seedling traits phenotyped under normal (S0) and water stress (S5) conditions**. **(A)**, root length (RL); **(B)**, shoot length (SL); **(C)**, root to shoot length ratio (R/S); **(D)**, fresh weight (FW).

Analysis of variance indicated significant differences among accessions, between environments and their interactions (*P* < 0.01). Significant differences were observed between the means for all traits under normal (S0) and water stress (S5) conditions. Under water stress, RL and R/S significantly increased by 14.3% and 91.0%, respectively, but SL and FW significantly decreased by 40.1% and 60.1%, respectively, compared to the control (Table 1). Medium to high heritability estimates were obtained for all tested traits, ranging from 68.2 (SL under S5) to 88.6 (R/S under S5). R/S showed high heritability (88.1 under S0 and 88.6 under S5) compared to RL (87.8 under S0 and 86.1 under S5) (Table 1). The level of phenotypic variation detected among all accessions was affected by water stress. Relative to the average variation of all measured traits under normal conditions, variation in RL and R/S increased while variation in SL and FW decreased under water stress conditions (Figure 1).

**Table 1:** **Mean values and broad-sense heritability of seedling traits observed under normal (S0) and water stress (S5) conditions**.

**Trait^a^**	**Treatment**	**Mean**	***SD***	**Reduction^b^(%)**	**Broad-sense heritability (h${}_{B}^{2}$)**	**Accessions (A) (139 d.f.)^c^**	**Treatments (T) (1 d.f.)**	**A × T (1112 d.f.)**
RL (cm)	S0	7.8	2.3	−14.3	87.8	^**^	^**^	^**^
	S5	8.9	2.8		86.1	^**^	^**^	^**^
SL (cm)	S0	4.7	1.1	40.1	77.7	^**^	^**^	^**^
	S5	2.8	0.7		68.2	^**^	^**^	^**^
R/S	S0	1.8	0.7	−91.0	88.1	^**^	^**^	^**^
	S5	3.4	1.8		88.6	^**^	^**^	^**^
FW (mg/seedling)	S0	147.5	37.4	60.1	86.5	^**^	^**^	^**^
	S5	58.9	20.8		83.4	^**^	^**^	^**^

a *RL, root length; SL, shoot length; R/S, root to shoot length ratio; FW, fresh weight*.

b $Reduction(\%)=\frac{\left(Trait\;under\;S0-Trait\;under\;S5\right)}{Trait\;under\;S0}\times 100\;\%.$

c d.f., degrees of freedom; ^**^Significant at the 1% level of significance among accessions (A), between treatments (T) and interaction effects of accessions by treatments (A × T).

### Correlation coefficients among seedling traits measured under normal (S0) and water stress (S5) conditions

All correlation coefficients for pairs of measured traits between control and water stress conditions were significant and positive (*P* < 0.01). R/S was significantly and positively correlated to RL, but negatively correlated to SL under both control and water stress conditions. FW was significantly and positively (*P* < 0.01) correlated to RL and R/S under both control and water stress conditions, but relatively lower correlation coefficients were observed in the control than under water stress. Also, significant and positive (*P* < 0.01) correlations were observed between FW and SL under water stress conditions. DSI was significantly and negatively correlated to SL and FW in the control, but positively correlated to RL, R/S and FW under water stress (Table 2).

**Table 2:** **Pearson's phenotypic correlation coefficients between seedling traits measured under normal (S0) and water stress (S5) conditions**.

**Trait^a^**	**RL (cm)**	**SL (cm)**	**R/S**	**FW (mg/seedling)**	**DSI (%)^b^**
RL (cm)	0.48^**^^c^	−0.12 ns	0.73^**^	0.56^**^	−0.13
SL (cm)	0.00 ns	0.65^**^	−0.69^**^	0.16 ns	−0.18^**^
R/S	0.72^**^	−0.57^**^	0.56^**^	0.23^**^	0.05
FW (mg/seedling)	0.68^**^	0.28^**^	0.33^**^	0.49^**^	−0.28^**^
DSI (%)	0.46^**^	0.14 ns	0.26^**^	0.72^**^	**NA**

*Data from water stress (S5, lower left) and normal (S0, upper right) conditions of 140 canola inbred lines and the cross correlation coefficients between S5 and S0 (diagonal in bold). Phenotypic correlation coefficients were calculated between the traits using means from five replications*.

a *RL, root length; SL, shoot length; R/S, root to shoot length ratio; FW, fresh weight; DSI, drought stress index*.

b $DSI=\frac{FW\;under\;S5}{FW\;under\;S0}\times 100\%$.

c ^*^ and ^**^ indicated the correlation coefficient was significant at the 5% and 1% level, respectively; ns indicated the correlation coefficient was not significant.

### Genome-wide association studies

**Figure 2:**
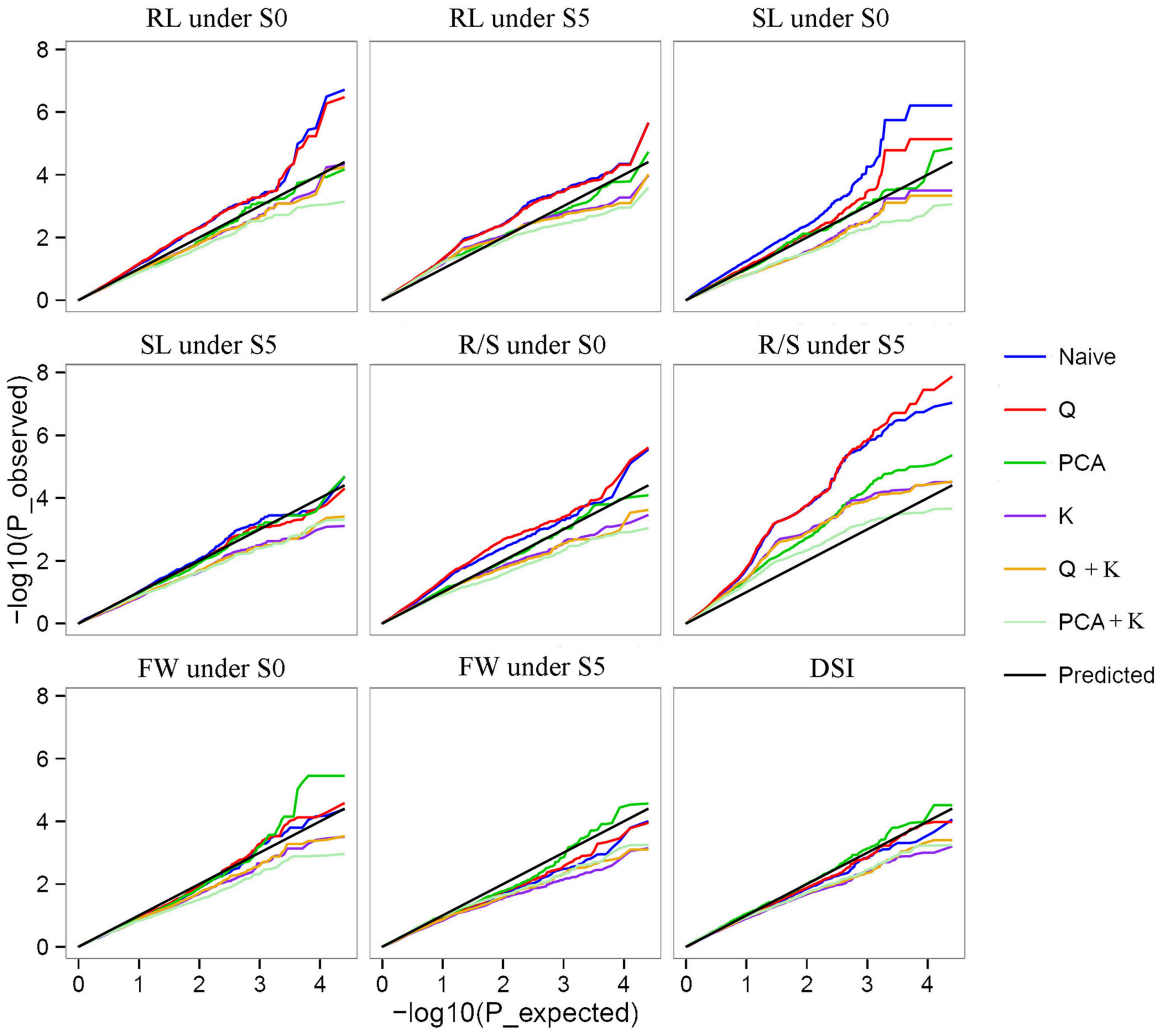
**Quantile-quantile plots of estimated −log_10_(p) from association analysis using six models for drought stress index (DSI) and four traits under normal (S0) and water stress (S5) conditions**. Root length (RL) under S0 and S5; Shoot length (SL) under S0 and S5; Root to shoot length ratio (R/S) under S0 and S5; Fresh weight (FW) under S0 and S5; Drought stress index (DSI).

**Figure 3:**
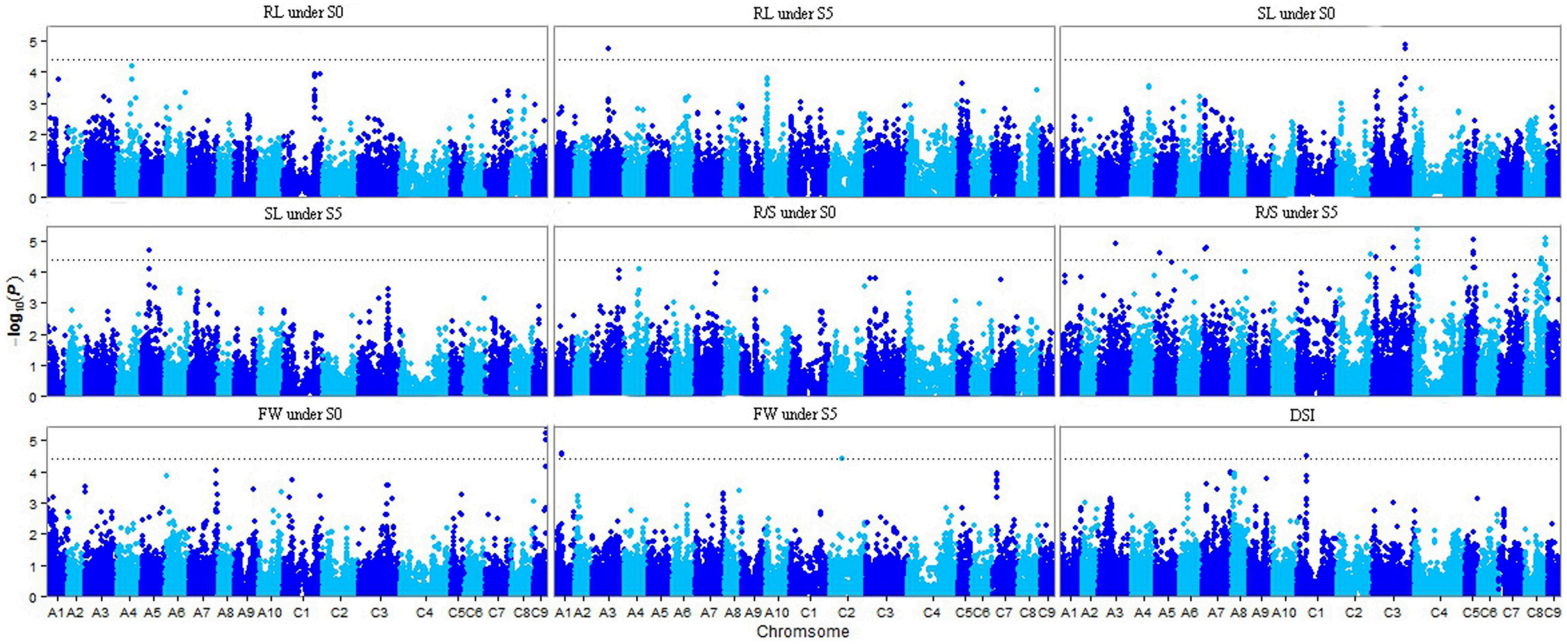
**Manhattan plots resulting from GWAS results for drought stress index and four traits under normal (S0) and water stress (S5) conditions**. Root length (RL) under S0 and S5; Shoot length (SL) under S0 and S5; Root to shoot length ratio (R/S) under S0 and S5; Fresh weight (FW) under S0 and S5; Drought stress index (DSI). The dashed horizontal line depicts the significance threshold (*P* < 3.92 × 10^−5^).

Of the six models tested, the PCA model fitted the data best for all measured traits except R/S under S5 and FW under S0 according to QQ plots (Figure 2). Therefore, the PCA model (generalized linear model (GLM) with 10 principal components for population structure) was used to perform GWAS and identify significant associations. A total of 36 SNP-trait associations with *P* < 3.92 × 10^−5^ were identified based on Manhattan plots (Figure 3); all significant SNPs within 200 kb were consolidated into one highest *P*-value SNP to account for linkage disequilibrium. Hence, a total of 17 associations involving 16 SNPs were identified, explaining 25.8–35.8% of the phenotypic variance (Table 3). However, more associations were identified under water stress than control conditions, and 10 associations were detected for R/S under S5 (Table 3).

**Table 3:** **Leading genome-wide association SNPs for seedling traits under normal (S0) and water stress (S5) conditions**.

**Trait^a^^,^^b^**	**SNP**	**Allele**	**Linkage group**	**Position**	**−log_10_(P)**	***R*^2^ (%)**	**Published drought-related genes or QTL**	**Position of published genes or QTL**
SL under S0	Bn-scaff_16394_2-p1222322	[A/G]	C3	50058482	4.85	26.66		
SL under S5	Bn-A05-p6415983	[A/G]	A5	5964030	4.68	25.77	BnPIP1 (Yu et al., 2005; Kagale et al., 2007)	A5:6368730
RL under S5, R/S under S5	Bn-A03-p15141821	[T/C]	A3	14250181	4.73, 4.90	29.72	GU189589 (Chen et al., 2009)	A3:13907353
R/S under S5	Bn-scaff_15908_1-p140050	[T/C]	A5	3535320	4.58	26.88	BNECP63 (Singh et al., 2009)	A5:4193606
	Bn-A10-p11466727	[A/C]	A7	2036064	4.73	29.49	BnaCPK18 (Zhang et al., 2014)	A7:2037903
	Bn-A10-p3063220	[T/C]	A7	4149628	4.79	33.46		
	Bn-scaff_17623_1-p657238	[T/C]	C2	42770184	4.56	26.80	BnPIP1 (Yu et al., 2005; Kagale et al., 2007)	C2:42871610
	Bn-scaff_22728_1-p1185330	[A/G]	C3	5479710	4.45	26.77	BNLAS (Yang et al., 2010)	C03:4993284
	Bn-scaff_17521_1-p1398928	[T/G]	C3	20851748	4.77	27.81	GU189589 (Chen et al., 2009)	C3:20465796
	Bn-scaff_19248_1-p73640	[T/C]	C4	6627161	5.37	31.29	GU189586 (Chen et al., 2009)	C4:6662462
	Bn-scaff_16268_1-p444284	[A/G]	C5	12805031	5.02	30.31	BnPIP 1(Yu et al., 2005; Kagale et al., 2007)	C5:12258275
	Bn-scaff_16445_1-p1834116	[A/G]	C8	34965909	5.08	34.32	GU189582 (Chen et al., 2009)	C8:34681768
FW under S0	Bn-scaff_16362_1-p404058	[T/C]	C9	43721409	5.45	33.17	BN28a (Lee et al., 2012)	C9:43714965
FW under S5	Bn-A01-p7558345	[A/G]	A1	6898964	4.57	31.09	*rsdw1.4* (Li et al., 2014b)	A1: 6971413
	Bn-scaff_20221_1-p84872	[A/G]	C2	24189052	4.44	35.77	BnaCPK17 (Zhang et al., 2014)	C2:23752130
DSI	Bn-scaff_17369_1-p625180	[T/C]	C1	11746556	4.52	28.93	BnPIP1 (Yu et al., 2005; Kagale et al., 2007)	C1:11562792

a *RL, root length; SL, shoot length; R/S, root to shoot length ratio; FW, fresh weight; DSI, drought stress index*.

b $DSI=\frac{FW\;under\;S5}{FW\;under\;S0}\times 100\%$.

### Exploration of degs in response to water stress

**Figure 4:**
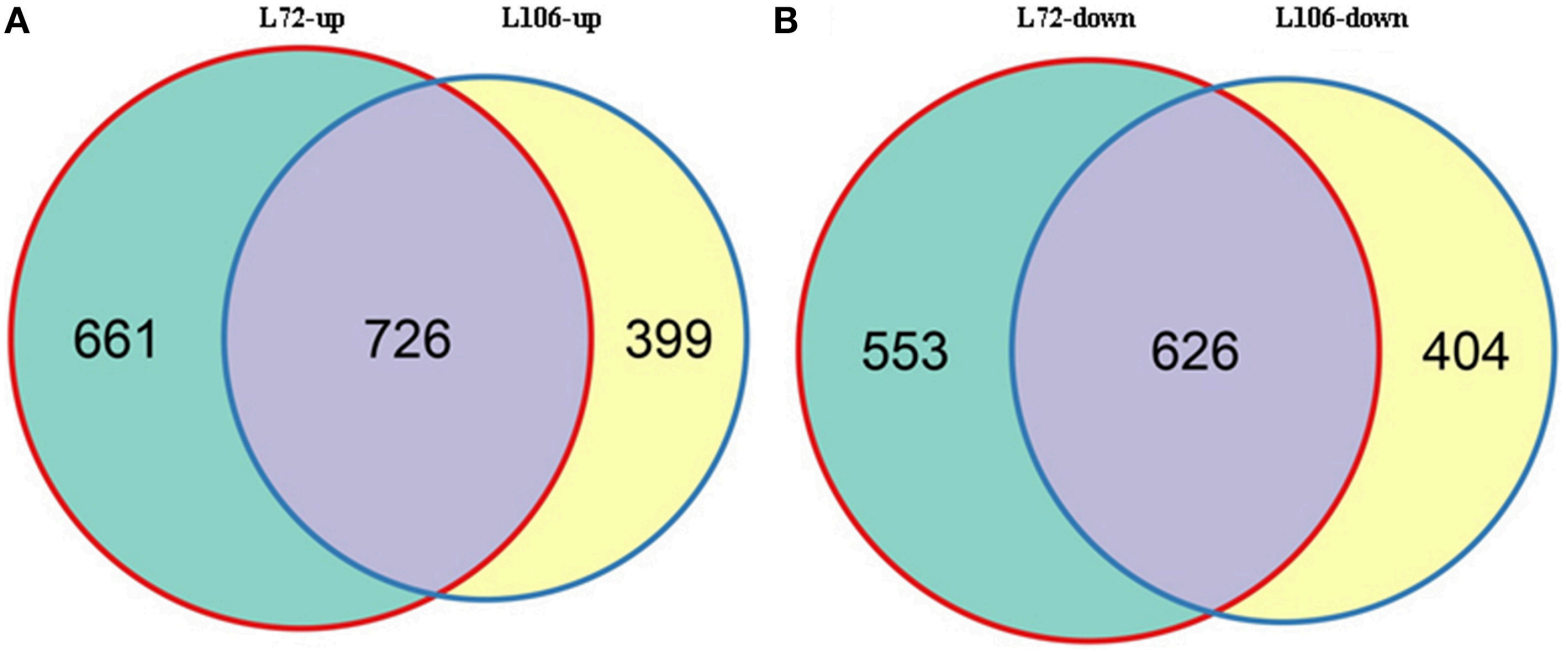
**Overlap between differentially-expressed genes (DEGs) in two *B. napus* accessions (L72: MUTU and L106: Alku)**. Venn diagrams display overlap between DEGs in L72 (green) and L106 (yellow) after exposure to −1.0 MPa (S10) water stress for 6 h. **(A)**, intersection of DEGs up-regulated in L72 with those up-regulated in L106; **(B)**, intersection of DEGs down-regulated in L72 with those down-regulated in L106.

A total of 2566 genes in L72 and 2155 genes in L106 were differentially regulated between water stress and control conditions. Of these, 1387 genes in L72 and 1125 genes in L106 were up-regulated and 1179 genes in L72 and 1030 genes in L106 were down-regulated (Table 4). A total of 726 up-regulated DEGs and 626 down-regulated DEGs were found in both L72 and L106 in the Venn diagram (Figure 4). Further analysis of these genes identified 21 drought-responsive genes in *B. napus* including five *BnLEA4* (Dalal et al., 2009) and five *BnECP63* (Singh et al., 2009) genes, which were significantly up-regulated in both accessions under water stress (Supplementary Table 3).

**Table 4:** **Differentially-expressed genes (DEGs) between seedlings under control (S0) and water stress (S10) conditions**.

**Lines**	**Treatments**	**Total clean reads**	**Mapped reads**	**Uniquely mapped reads**	**Number of water stress-induced genes**				
					**Total**	**Up-regulated**		**Down-regulated**	
						**Known^a^**	**Unknown^b^**	**Known**	**Unknown**
L72	S0	12583612	11791063 (93.7%)	8175037 (65.0%)	2566	1218	169	1078	101
	S10	12331549	11531448 (93.5%)	8021295 (65.0%)					
L106	S0	11656594	10926990 (93.7%)	7585794 (65.1%)	2155	972	153	960	70
	S10	12484808	11575069 (92.7%)	8145137 (65.2%)					

a *Known genes had Gene Ontology annotation*.

b *Unknown genes had no Gene Ontology annotation*.

*http://www.ncbi.nlm.nih.gov/protein/; http://www.ebi.ac.uk/interpro/*.

### Candidate drought-response genes

Based on both GWAS and RNA-seq analyses, 79 DEGs in both cultivars were identified within the vicinity of 1.0 Mb of the 16 SNP locations. Of these, 77 genes were homologs of 64 *A. thaliana* genes, with 58/64 annotated. There were 42 up-regulated genes and 37 down-regulated genes (Supplementary Table 4).

**Figure 5:**
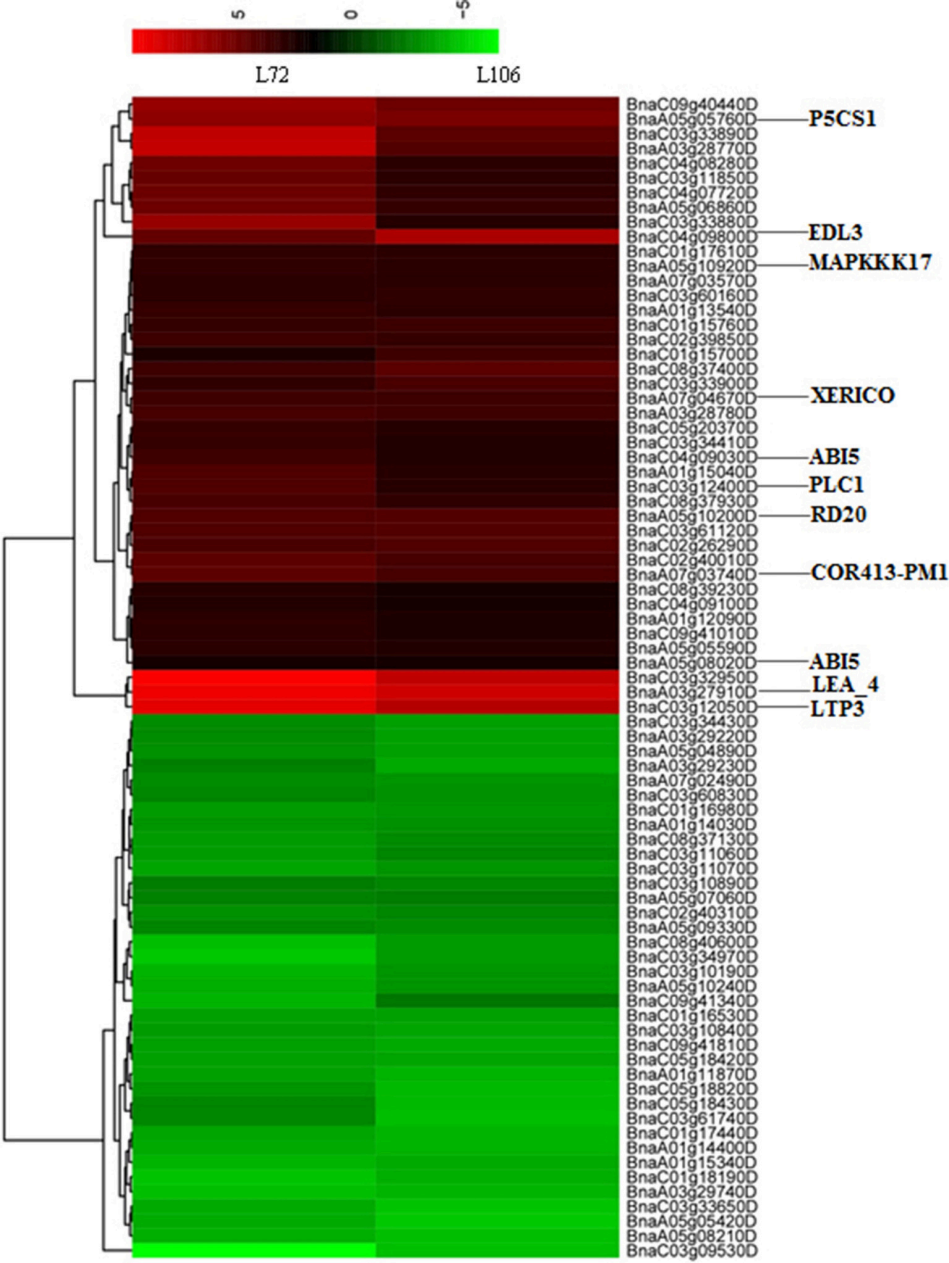
**Heatmap with hierarchical clustering showing differentially-expressed genes (DEGs) in two *B. napus* accessions (L72: MUTU and L106: Alku) within the vicinity of 1.0 Mb of 16 uniquely identified SNPs associated with water stress tolerance phenotypes**. The genes which were orthologs to drought-related genes in *A. thaliana* are shown.

In this study, 11 genes were homologs of annotated drought-related genes in *A. thaliana*, matched with six RL, SL and R/S trait-associated genomic regions under water stress. Moreover, these 11 genes were up-regulated in both tested accessions (Figure 5). One gene, *BnaA03g27910D*, was located 568 kb from the marker Bn-A03-p15141821 (associated with both RL under S5 and R/S under S5) and encoded for an ortholog of *A. thaliana* gene “Late embryogenesis abundant protein (LEA) family protein (*LEA4*)”; two genes, *BnaA05g10200D* and *BnaA05g10920D*, were located 405 and 33 kb from the marker Bn-A05-p6415983 (associated with SL under S5) and encoded orthologs to *A. thaliana* genes “mitogen-activated protein kinase kinase kinase 17 (MAPKKK17)” and “Caleosin-related family protein (*RD20*),” respectively; *BnaA05g05760D* was located 495 kb from marker Bn-scaff_15908_1-p140050, and encoded for ortholog to *A. thaliana* gene “delta1-pyrroline-5-carboxylate synthase 1 (*P5CS1*)”; *BnaA07g03740D* and *BnaA07g04670D* were 760 and 928 kb from SNP marker Bn-A10-p3063220, encoded for orthologs to *A. thaliana* genes “cold regulated 413 plasma membrane 1(*COR413PM1*)” and “RING/U-box superfamily protein (*XERICO*),” respectively; *BnaC03g12050D* and *BnaC03g12400D* were located 362 and 513 kb from marker Bn-scaff_22728_1-p1185330, and encoded for orthologs of *A. thaliana* genes “lipid transfer protein 3 (*LTP3*)” and “phospholipase C1 (*PLC1*),” both induced by dehydration stress; and *BnaA05g08020D* was 878 kb from Bn_scaff_15908_1-p140050 (associated with R/S under S5) and *BnaC04g09030D* was 148 kb from Bn-scaff_19248_1-p73640 (associated with R/S under S5), which both encoded for orthologs of *A. thaliana* genes “Basic-leucine zipper (bZIP) transcription factor family protein (ABI5)” (Supplementary Table 4).

### Comparisons of candidate drought-response genes using qRT-PCR with RNA-seq

**Figure 6:**
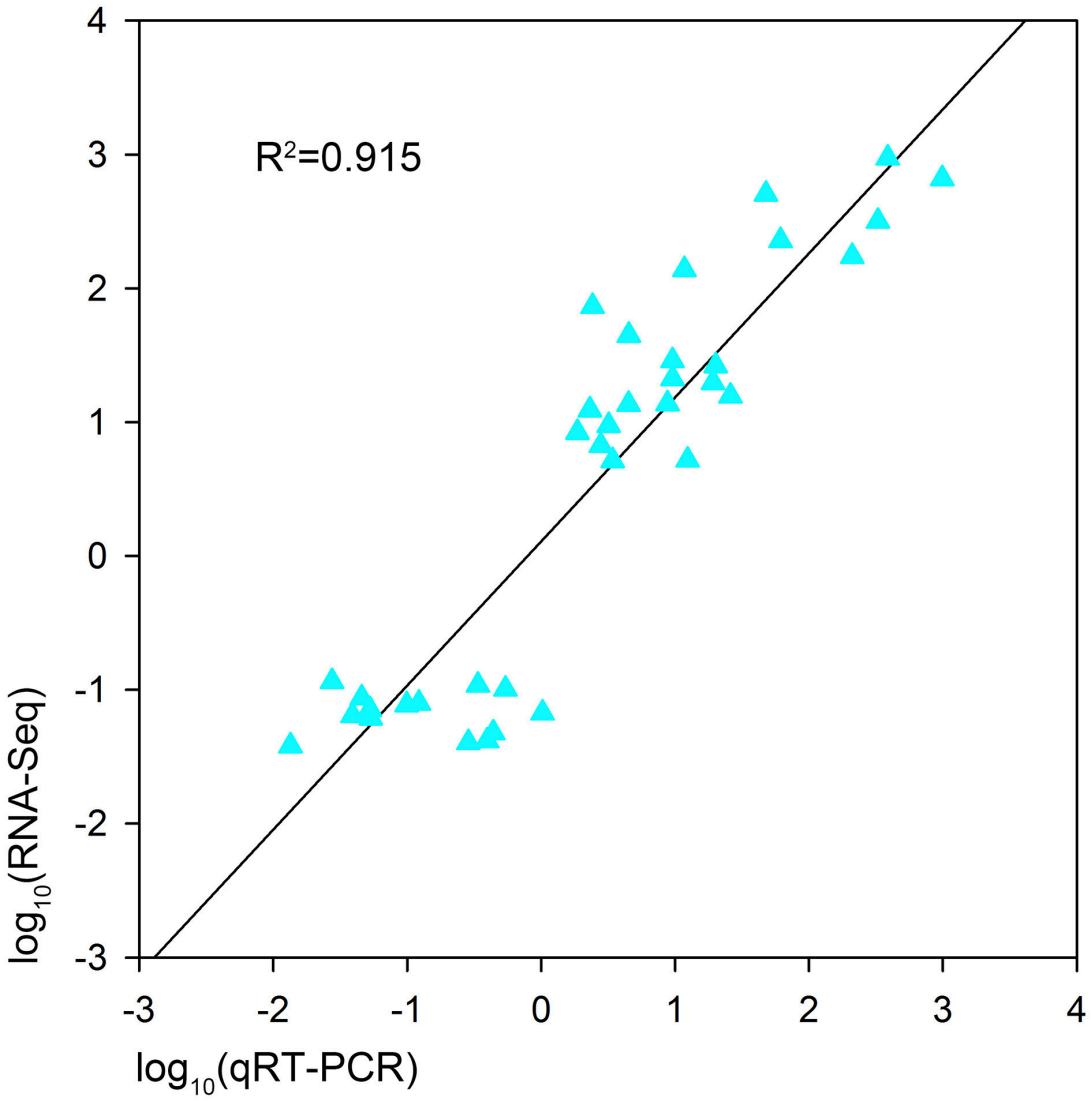
**Pearson's correlation between the expression profiles of 18 genes by qRT-PCR and RNA sequencing (RNA-seq) in two *B. napus* accessions (L72: MUTU and L106: Alku)**. A significant positive correlation is shown (*R*^2^ = 0.915, *P* < 0.01).

**Figure 7:**
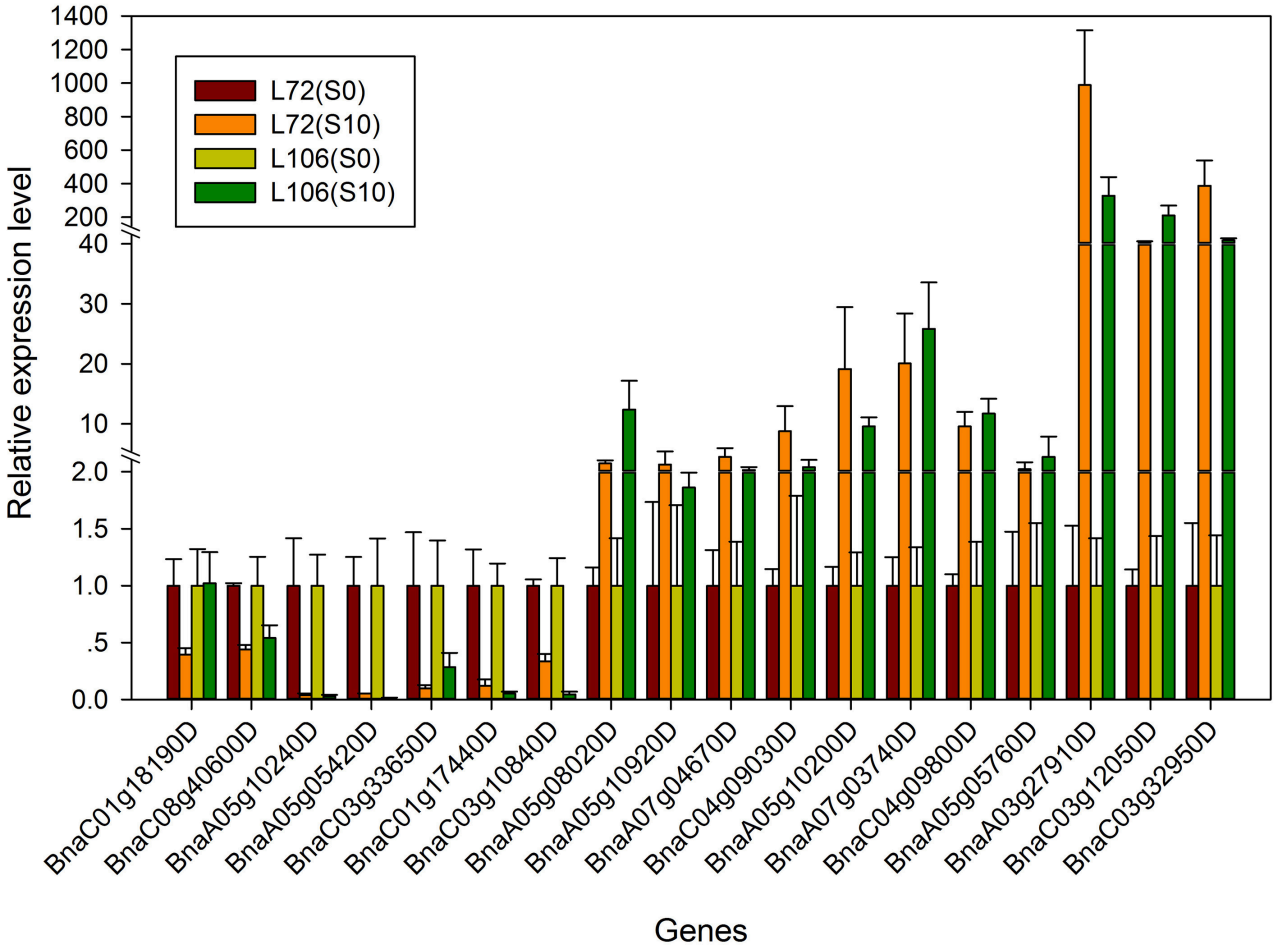
**The relative expression level of the 18 genes from real-time quantitative PCR (qRT-PCR) in two *B. napus* accessions (L72: MUTU and L106: Alku)**. S10, seedlings sampled after exposure to −1.0 MPa water stress for 6 h; S0, seedlings sampled in the parallel control condition.

In order to validate the expression profiles from RNA-seq, qRT-PCR was performed with 18 genes which were orthologs to drought responsive genes in *A. thaliana*. The Pearson's correlation coefficient of genes expression level between the two platforms was calculated. It showed that the gene expression level using RNA-seq was significantly (*R*^2^ = 0.915, *P* < 0.01) correlated with those using qRT-PCR (Figure 6). The validated genes included 8 down-regulated genes and 10 up-regulated genes, most of the genes showed consistent expression profiles with those from RNA-seq (Figure 7). The results suggested that combined GWAS with RNA-seq was an accurate and reliable way to screen for water stress tolerant candidate genes in canola.

## Discussion

### Index of seedling water stress tolerance

Establishing an index of water stress tolerance under water stress conditions would facilitate population screening and breeding. In this study, it was noted that R/S was sensitive to water stress as reflected by the 91.0% increment, much higher variation level and relatively high broad-sense heritability (h${}_{\text{B}}^{2}$) under water stress (Table 1 and Figure 1). This result was in line with findings in wheat, where the authors suggested that selection on the basis of root to shoot length ratio could be beneficial (Dhanda et al., 2004). In addition, R/S showed much higher correlations with FW and DSI under drought conditions than those under control conditions (Table 2). It is possible that the plants avoided low water potential by balancing water uptake and water loss, while the continued root growth under water stress was important for water uptake and the shorter (smaller) plants resulted in less water loss (Dhanda et al., 2004; Ruta et al., 2010). Therefore, plants with relatively higher R/S may have relatively higher FW and DSI under water stress. More significant associations were observed for R/S under water stress conditions than in the normal. Therefore, R/S could be used as a reliable indicator for evaluating water stress tolerance at early seedling growth stages.

### Genome-wide association of SNPs with drought tolerance

With the rapid development of new technologies in DNA sequencing and bioinformatics, and reduction in associated costs, GWAS are rapidly becoming a standard tool for detecting natural variation which accounts for complex quantitative phenotypes in plants (Li et al., 2013). Mixed linear models (MLM) are popular for detecting genotype–phenotype associations in plant GWAS, but are more stringent than GLMs and can result in type II errors (false negatives), while naïve GLMs can result in type I errors (false positives) (Pace et al., 2015). In this study, observed *P*-values fitted better to the expected *P*-values using the PCA model than other models, indicating that population structure correction in a PCA model was more efficient at removing false positives than the STRUCTURE algorithm (Figure 2) (Montilla-Bascón et al., 2015). The PCA model has been used to dissect nine agronomic traits in maize under well-watered and water-stressed conditions (Xue et al., 2013).

Although only 66 lines were used in the association panel in our study, these lines showed consistent water stress tolerant (16), moderate (34) and water stress sensitive (16) phenotypes. Of the 16 SNP loci associated with one or two phenotyped traits, some were located close to previously identified drought-responsive genes. For example, 10 drought-related QTLs or genes reported previously were close to 14 of the significantly associated SNPs we identified. Bn-A05-p6415983 associated with SL under S5 was 406 kb from *BnPIP1* (Yu et al., 2005; Kagale et al., 2007) and Bn-A03-P15141821 associated with both R/S under S5 and RL under S5 was 343 kb from drought-responsive genes-*GU189589* (Chen et al., 2009). Of the nine SNPs associated with R/S under S5, eight were 2–658 kb from published drought-related genes (Yu et al., 2005; Kagale et al., 2007; Chen et al., 2009; Singh et al., 2009; Yang et al., 2010; Zhang et al., 2014). Bn-scaff_16362_1-p404058 associated with FW under S0 was only 6 kb from *BN28a* (Lee et al., 2012); Bn-A01-p7558345 and Bn-scaff_20221_1-p84872 associated with FW under S5 were 72 and 437 kb from the reported drought-related QTL-*rsdw1.4* and gene-*BnaCPK17*, respectively (Li et al., 2014b; Zhang et al., 2014). Bn-scaff_17369_1-p625180 associated with DSI was 184 kb from *BnPIP1* (Yu et al., 2005; Kagale et al., 2007; Table 3). These results support the success of the association genetics approach used in this study.

### Details of candidate genes

For the DEGs in response to water stress, 40% of total genes in the two lines followed a similar pattern (Figure 4) while 60% of the total genes followed different patterns. This outcome may be due to the differed genetic backgroud of the two lines since they are originated from two different sources, one from Australia while the other from Finland. Their different responses to nutrients (the seedlings were exposed to ¼ strength Hoagland's solution supplemented with PEG 6000) may also result in DEGs (Figure 4). In order to exclude the above effects, the differentially expressed genes overlapped in both lines would be plausible to detect the drought responding genes. We therefore, combined GWAS with DEGs expressed in both tested lines to identify water stress tolerant candidate genes in *B. napus*.

Under drought, R/S and RL were strongly correlated with DSI, an indicator of drought tolerance (Kpoghomou et al., 1990). Thus, the SNP marker Bn-A03-p15141821, associated with both R/S and RL, is of particular interest. The gene, *BnaA03g27910D*, is located near SNP marker Bn-A03-p15141821 and encodes for an ortholog of *A. thaliana* gene *LEA4*. LEA proteins are hydrophilic, mostly intrinsically-disordered proteins, which play major roles in desiccation tolerance (Hundertmark and Hincha, 2008; Candat et al., 2014). Enhanced expression of LEA genes is thought to contribute to desiccation tolerance (Jakoby et al., 2002). Furthermore, transgenic *Arabidopsis* plants over-expressing *BnLEA4-1* showed enhanced tolerance to drought stress (Dalal et al., 2009). In this study, the expression level of gene-*BnaA03g27910D* was greatly up-regulated in both accessions tested. Hence, *BnaA03g27910D* is a plausible candidate gene for drought tolerance in *B. napus*.

This study suggests that R/S could be used as an index of drought tolerance under drought treatments, and R/S was involved in 10 of 16 identified marker-trait associations. Based on this approach, six particularly promising candidate genes located near four SNP markers associated with R/S were identified. The gene-*BnaA05g05760D* encoded for ortholog of *A. thaliana* gene *P5CS1*. *P5CS1* encodes the delta1-pyrroline-5-carboxylate synthase enzyme which catalyzes the rate-limiting step of proline biosynthesis (Székely et al., 2008). Enhanced expression of *P5CS1* was observed in both *B. napus* accessions under water stress, and may lead to augmented proline synthesis. Proline accumulation is believed to play a role in plant stress tolerance, and elevated proline levels may contribute to drought tolerance (Verbruggen and Hermans, 2008; Sharma and Verslues, 2010). *BnaA07g03740D* and *BnaA07g04670D* close to SNP marker Bn-A10-p3063220, encoded orthologs to *A. thaliana* genes *COR413PM1* and *XERICO*. *COR413PM1* is a stress-inducible gene, known to be up-regulated in response to cold and desiccation (Huang et al., 2008; Fang et al., 2015). Previous studies have indicated that up-regulation of *XERICO* confers drought tolerance in *A. thaliana* (Ko et al., 2006). In this study, the two genes were up-regulated under water stress, which was consistent with previous research. Another two genes-*BnaC03g12050D* and *BnaC03g12400D*, located near marker Bn-scaff_22728_1-p1185330, encode orthologs of *A. thaliana* genes *LTP3* and *PLC1* which were both induced by dehydration stress (Hirayama et al., 1995; Guo et al., 2013). The overexpression of *LTP3* and *PLC1* enhanced drought tolerance in *A. thaliana* and maize, respectively (Wang et al., 2008; Guo et al., 2013). In this study, *BnaC03g12050D* and *BnaC03g12400D* significantly enhanced expression under water stress. *BnaC04g09800D*, close to marker Bn-scaff_19248_1-p73640,encoded *EDL3* which is an F-box protein involved in the regulation of abscisic acid signaling, and strong and rapid induction of *EDL3* gene expression was observed under osmotic stress in *A. thaliana*, in line with the results in this study (Koops et al., 2011). In addition, enhanced expression of *BnaA05g10200D* under water stress was observed in this study. *BnaA05g10200D* is located 405 kb from the marker Bn-A05-p6415983 (associated with SL under S5), and encodes *RD20* which plays a role in drought tolerance through stomatal control under water deficit conditions (Aubert et al., 2010). Hence, *BnaA05g10200D* may be considered a possible candidate gene for drought tolerance.

Apart from the above eight genes which were orthologs to drought tolerant genes of *A. thaliana*. We found that *BnaC03g32950D* is located 684 kb from the marker Bn-scaff_17521_1-p1398928 (associated with R/S under S5) and clustered to the same category as *BnaA03g27910D and BnaC03g12050D* (Figure 5). Also, its expression level under water stress condition was significantly up-regulated in both tested lines. Therefore, we deduced that it may be a possible new candidate gene for water stress tolerance in *B. napus* not characterized in other plants as for water stress tolerance. Further investigation and validation is needed to confirm its function in water stress tolerance in *B. napu*s.

In conclusion, this study supports the use of the root to shoot length ratio as a reliable indicator for drought tolerance at early seedling growth stages. To our knowledge, this is the first report for a genome-wide association of drought tolerance using the Illumina Brassica 60K SNP array in canola for the detection of 16 significantly associated loci. By combining the significantly associated loci and DEGs identified by RNA-seq, 79 candidate genes were identified. Of these genes, nine may be strongly associated with water stress tolerance. Further investigation and validation of these genes is underway to confirm their function in the tolerance of *B. napus* to water stress.

## Author contributions

GY, JZ, and LH designed the study. JZ and XZ carried out the experiment work for phenotyping. AM performed the SNP genotyping and filtering. JZ performed the DNA and RNA extractions. JZ, JW, SL, and TL did the data analysis. BR contributed most of the seed materials. JZ wrote the manuscript. AM, GY, JB, BR, and LH critically revised the manuscript. All authors have read and approved the final version of the manuscript.

### Conflict of interest statement

The authors declare that the research was conducted in the absence of any commercial or financial relationships that could be construed as a potential conflict of interest.
